# Nondestructive 3D Imaging of Microscale Damage inside Polymers—Based on the Discovery of Self‐Excited Fluorescence Effect Induced by Electrical Field

**DOI:** 10.1002/advs.202302262

**Published:** 2023-06-28

**Authors:** Wenxia Sima, Xinyu Tang, Potao Sun, Zhenkun Sun, Tao Yuan, Ming Yang, Chun Zhu, Zeyan Shi, Qin Deng

**Affiliations:** ^1^ State Key Laboratory of Power Transmission Equipment and System Security and New Technology Chongqing University Chongqing 400044 China; ^2^ Key Laboratory of Energy Thermal Conversion and Control Ministry of Education School of Energy and Environment Southeast University Nanjing 210096 China; ^3^ Analytical and Testing Center Chongqing University Chongqing 400030 China

**Keywords:** 3D reconstruction, electrical tree, fluorescence self‐excitation, non‐destructive imaging, silicone gel

## Abstract

The development of high‐precision, non‐destructive, and three‐dimensional (3D) in situ imaging of micro‐scale damage inside polymers is extremely challenging. Recent reports suggest that 3D imaging technology based on micro‐CT technology causes irreversible damage to materials and is ineffective for many elastomeric materials. In this study, it is discovered that electrical trees inside silicone gel induced by an applied electric field can induce a self‐excited fluorescence effect. Based on this, high‐precision, non‐destructive, and 3D in situ fluorescence imaging of polymer damages is successfully achieved. Compared with the current methods, the fluorescence microscopic imaging method enables slicing of the sample in vivo with high‐precision operation, realizing the precise positioning of the damaged area. This pioneering discovery paves the way for high‐precision, non‐destructive, and 3D in situ imaging of polymer internal damage, which can solve the problem of internal damage imaging in insulating materials and precision instruments.

## Graphical abstract

This study reports for the first time the characterization of electrical tree degradation in polymers through the detection of self‐excited fluorescence in the channel area of the tree. Furthermore, this high‐precision, in situ technique is developed without causing further damage to the polymer which is an issue that is present in currently used imaging techniques.

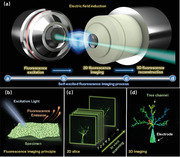

## 1 Introduction

High‐precision, 3D imaging of micro‐scale damage inside polymers has long been interesting but challenging work and is of great significance for the efficient discovery of internal defects in polymers and analysis of material failure mechanisms.^[^ ^1^, ^2^, ^3^ ^]^ However, currently there is a significant lack of high‐precision, non‐destructive, and in situ 3D imaging technology for assessing the internal micro‐scale damages to insulating materials when operating under strong electrical fields and complex mechanical stress.^[^ ^4^, ^5^ ^]^ Consequently, the development of basic scientific research regarding insulating materials for electrical equipment and electronic devices is greatly restricted.

Electrical tree degradation is a form of micro‐scale damage that occurs inside polymers when subjected to a high local electrical field.^[^ ^6^, ^7^, ^8^ ^]^ The electrical tree structure (Figure S1, Supporting Information), named so owing to its tree‐like shape, is formed by local split channels several micrometers in width.^[^ ^9^, ^10^ ^]^ The most used method for imaging the damage inside electrical tree structures is obtaining the projection of the damage morphology in the imaging plane through an optical microscope^[^ ^11^, ^12^, ^13^ ^]^ or CCD camera.^[^ ^14^, ^15^, ^16^ ^]^ Although these methods can capture the macroscopic information of the damage in two–dimensional (2D) scale, such as the length and morphological characteristics of the electrical tree, the spatial information of the damaged area is inevitably lost in the planar imaging method. Therefore, conventional imaging methods cannot accurately reflect the actual morphology of the electrical tree, and for most opaque insulation materials, these methods almost always fail.

**Scheme 1:**
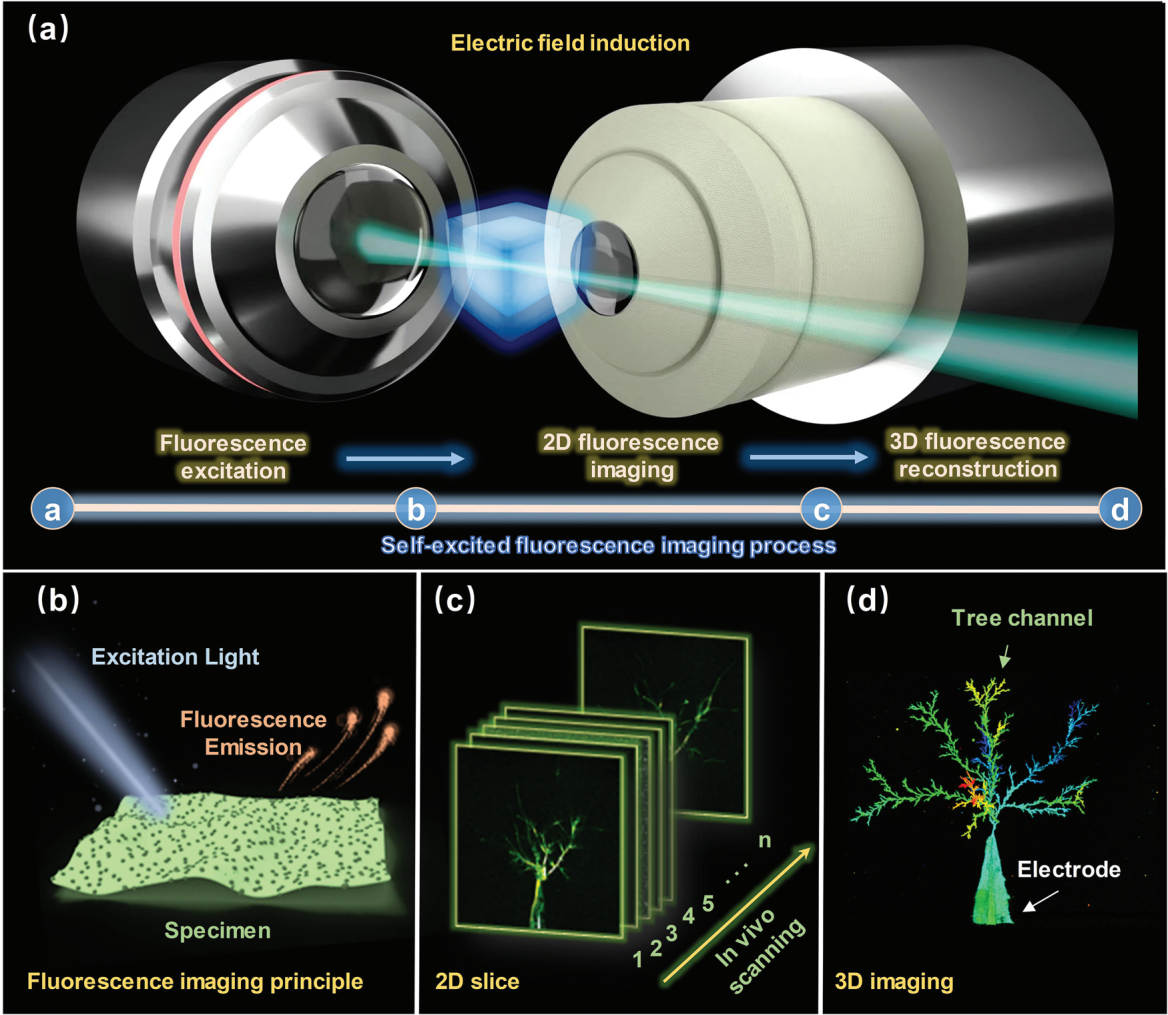
Self‐excited fluorescence imaging process. a) Fluorescence microscopic system. b) Fluorescence imaging principle. c) In vivo scanning 2D slice. d) Electrical tree morphology 3D reconstruction.

In recent years, X‐ray computed tomography technology (XCT) has been frequently used to perform 3D imaging of internal damage in materials.^[^ ^17^, ^18^, ^19^ ^]^ Compared with 2D imaging, 3D imaging is superior for visualizing the complexity of the damage channels. Although XCT imaging method can elucidate the 3D characteristics of the internal damage to a certain extent, it still cannot directly image the damaged area,^[^ ^20^, ^21^ ^]^ and so, the sample needs to be physically sliced, which leads to slice deformation and renders the dataset difficult to register.^[^ ^22^, ^23^, ^24^ ^]^ These errors can have an impact on the final back‐calculation and image process,^[^ ^19^, ^25^, ^26^, ^27^, ^28^, ^29^ ^]^ causing discrepancies between the imaging results and the actual 3D electrical tree morphology. More importantly, XCT produces high‐temperature radiation damage and vacuum stress to materials during the imaging process,^[^ ^18^ ^][^ ^30^, ^31^ ^]^ which is problematic for electronic devices requiring extremely stable operating environments. Furthermore, XCT technology is mostly suited to epoxy resin,^[^ ^32^, ^33^ ^]^ as opposed to elastic insulating materials, such as silicone gel. Therefore, it is necessary to develop a novel imaging method to obtain the damage morphology inside precision instruments (**Scheme** **1**).

In our previous studies, it was incidentally found that under the induction of an electric field, electrical tree channels inside silicone gel can excite fluorescence, otherwise known as the self‐excited fluorescence effect. More interestingly, the fluorescence intensity reportedly corresponds to the intensity of the damage and the structure of the channels. Inspired by this phenomenon, this study proposes a novel 3D imaging method based on in situ self‐excited fluorescence of micro‐scale damage inside polymers. The method utilizes the strong electrical field inside the silicone gel to induce electrical tree degradation and self‐excited fluorescence effect and combines a 3D in vivo scanning mechanism to reveal the precise 3D morphology of the complex micro‐scale structure in the material without causing any damage. To the best of our knowledge, this is the first time that 3D imaging of micro‐scale structures has been investigated using self‐excited fluorescence. This technology has potential applications such as non‐destructive, in situ, and high‐precision damage detection.

## 2 Results and Discussion

### 2.1 The Discovery of Self‐Excited Fluorescence Phenomenon

**Figure 1:**
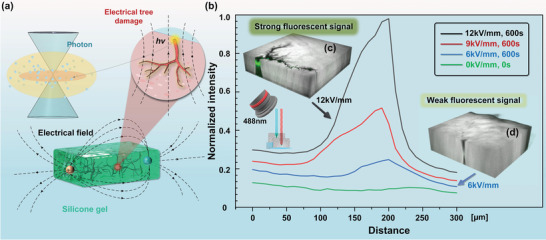
Schematic illustration of fluorescence imaging. a) Tree channel self‐excited fluorescence process when applied with an electrical field. b) The normalized curve of the tree channel fluorescence intensity when applied with varying electric field strengths. c) Tree channel morphology when the applied electrical field strength is 12 kV mm^−1^, and the electrical field action time is 600 s. d) Tree channel morphology when the applied electrical field strength is 6 kV mm^−1^, and the electrical field action time is 1000 s.

In this study, we found that the self‐excited fluorescence phenomenon occurred when the electrical split‐channel formed in the dielectric polymer under the action of an applied electrical field. To verify this phenomenon, we applied external electrical fields of varying intensities to the silicone gel material until electrical trees formed inside the polymer (Figure S5, Supporting Information). After this, fluorescence imaging was performed, and fluorescence intensity of the samples was measured. It was observed that the self‐excited fluorescence effect appeared within the electrical tree area inside the polymer. As shown in **Figure** **1**, fluorescence imaging of the electrical tree area was performed using excitation of a laser with a wavelength of 488 nm (Figure S4, Supporting Information). Figure 1b indicates that the fluorescence intensities induced by different electric field intensities were normalized and comparable. Once the electrical tree channel occurred, the fluorescence signal demonstrated an increasing trend with an increase in electrical field intensity. Furthermore, when the applied electrical field strength was 12 kV mm^−1^ and the electrical field action time was 600 s, the electrical tree channels were clearly observed in the polymer sample, and the corresponding fluorescence signal was the strongest. At this time, the fluorescence image was clearly observed from the result, as shown in Figure 1c,d demonstrates that electrical tree channel formation also occurred inside the polymer sample when the electrical field strength was only 6 kV mm^−1^, but the electrical field action time was 1000 s. However, the tree channel propagation was lower, and the corresponding fluorescence signal was also weaker.

**Figure 2:**
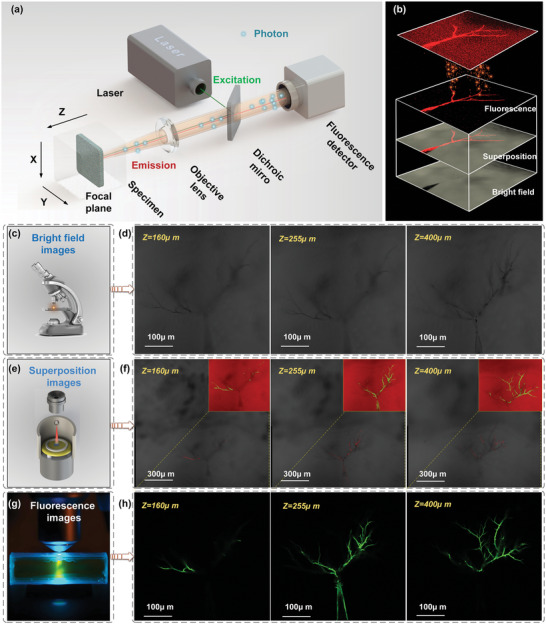
Schematic illustration of electrical tree fluorescence imaging. a) Imaging principle of confocal laser microscopic. b) Schematic illustration of electrical tree fluorescence imaging. c,d) Bright‐field imaging mode, *Z*‐axis depth are 160, 255, and 400 µm, respectively. e,f) Bright‐field and fluorescence superposition imaging mode, *Z*‐axis depth is 160, 255, and 400 µm, respectively. g,h) 488 nm fluorescence imaging mode, *Z*‐axis depth is 160, 255, and 400 µm, respectively.

In light of this, a new method was developed for the detection of internal damage in dielectric polymers by fluorescence microscopic imaging. The technology used a laser as the light source and adopted the coupled focusing technology of the light source pinhole and the detection pinhole to perform tomography on the sample to obtain a high‐resolution optical slice of the fluorescence microscopic system. Furthermore, the laser confocal microscopic imaging system could reach *Z*‐axis resolution,^[^ ^34^ ^]^ which allowed for non‐destructive optical sectioning of the sample along the *Z*‐axis to achieve imaging of the sample, and in situ imaging of electrical tree formation inside the polymers (**Figure** **2**a and Movie S1, Supporting Information). The confocal method utilized the location difference between the focused and defocused light on the optical path, whereby the defocused light was intercepted while the focused light was transmitted to the detection system. When the laser beam reached the tree channel, the molecules with fluorescent properties transitioned from the ground state to the excited state. The excited molecules then returned to the ground state through spontaneous radiation, simultaneously releasing fluorescent photons, which generated fluorescent signals (Figure 2b).

Simultaneously, the laser confocal microscope imaging system was used to scan the sample in the *XY* direction for *n* times, and capture optical sections along the *Z*‐axis direction of which the pixels in the *X*, *Y*, and *Z* directions were 1.5, 1.5, and 2 µm, respectively, and the scanning frequency was 400 Hz. Figure 2 indicates optical slices of various imaging modalities with varying focal planes (160 µm, 255 µm, 400 µm). Figure 2c,d depicts the imaging under bright‐field, which highlights the projection of all fields of view from the top of the sample to the focal plane. No significant difference existed in the imaging of different focal planes under the bright‐field imaging mode, indicating that this mode cannot accurately reflect the true morphology of the internal damage of the polymer. Superimposed imaging of bright‐field mode and fluorescence technology demonstrates that the fluorescence signal appears in different positions of the electrical tree channel under different focal planes (Figure 2e,f). Furthermore, it can also be observed from the fluorescence imaging that the intensity of the fluorescence signal and the imaging morphology were altered under different focal planes (Figure 2g,h). The comparison of different imaging modes highlights that the fluorescence imaging method can obtain the *Z*‐axis information of the imaging area, which provides more accurate spatial information for 3D imaging. With advantages of high definition and accurate positioning, the fluorescence imaging method has the ability to non‐destructively image the complex tomographic structure inside the polymer. The combination of bright field mode and fluorescence imaging mode not only proves that fluorescence signals appear at different positions in the damaged area, but also reflects the advantages of fluorescence imaging methods in live sectioning. In addition, the accuracy of the fluorescence imaging method was also demonstrated by filling the bright field imaging area with fluorescence signals.

### 2.2 Imaging of Fluorescence Self‐Excitation

A significant advantage of this imaging method is that it is non‐destructive, in vivo, section imaging. The laser scanning method can perform fluorescence imaging on the electrical tree of different focal planes, and the development and distribution of electrical trees within the polymer can be observed more thoroughly. This method illustrates the tomography of internal polymer damage, resolves the issue regarding low resolution in the imaging of thick samples, and avoids damage to the sample caused by traditional 3D imaging mechanical cutting.

**Figure 3:**
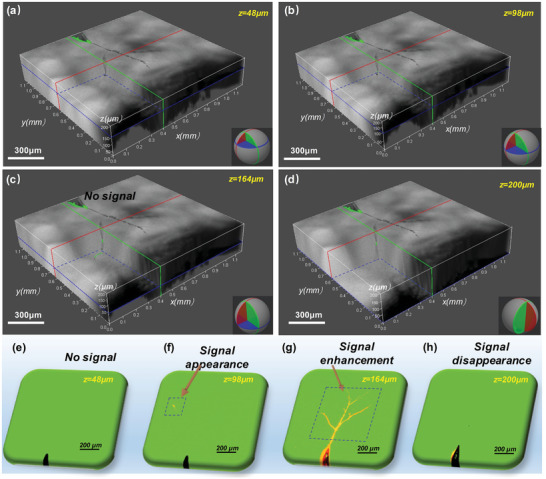
Fluorescence signal localization map of the electrical tree. a) *z* = 48 µm, no fluorescence signal is observed. b) *z* = 98 µm, fluorescence signal is observed. c) *z* = 164 µm, fluorescence signal can be clearly observed. d) *z* = 200 µm. No fluorescent signal is observed. (e–h) corresponds to (a–d) 2D in vivo fluorescence optical sections at *Z*‐axis depths.

**Figure** **3** and Figure S6, Supporting Information, present the cross‐sectional and in vivo section viewpoints of the superimposed 3D view of the bright‐field and fluorescence imaging of the electrical tree inside the polymer. Figure 3e–h depicts the in vivo fluorescence optical section at the *Z*‐axis depth corresponding to that of Figure 3a–d, which shows the fluorescence signals in the test sample from appearance to disappearance. Figure 3a depicts a 3D view of 48 µm depth, which means the Z–axis of this section image is 48 µm. No fluorescence signal was found at this depth, and although the bright‐field image projection of the electrical tree can be observed, no electrical tree exists inside the polymer. The fluorescence signal can be observed at 98 µm, and the fluorescence signal becomes increasingly stronger with increasing *Z*‐axis depth. When the *Z*‐axis depth reaches 200 µm, the fluorescence signal disappears. Therefore, the damage can be localized by the fluorescence laser confocal microscopic system to provide scale information for the damage extent of the electrical trees.

Figure S7, Supporting Information, shows the development process of the fluorescence signal of the 2D optical section from the appearance to the disappearance. The difference in the intensity of the fluorescence signal is displayed through the optical section of different depths of the focal plane. These light slices reveal the whole process of electrical tree imaging through the spatial information contained in the fluorescent signal, and demonstrate that the method of confocal fluorescence microscopic imaging can accurately locate the electrical tree inside the polymer without secondary damage to the materials.

**Figure 4:**
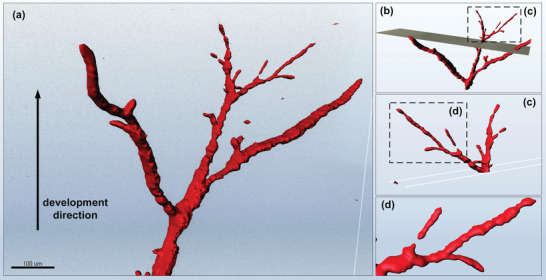
Schematic diagram of local channel reconstruction of electrical tree. a) 3D surface image of fluorescence imaging of electrical tree channel. The development direction is shown by the arrow. b) A cross‐section perpendicular to the development direction of electrical tree is set, this cross‐section can be set a study area of interest. c) Enlarged view of the boxed area in (b), the tree at the top splits to form two branches at different angles to the direction of the electric field. d) Enlarged view of the boxed area in (c), showing a pre‐discharge structure.

When the focal plane is adjusted and the sample is moved through the volume light sheet, the resulting fluorescence signal is recorded as a series of 2D images. 3D in situ imaging based on material fluorescence self‐excitation effect can be obtained by sequential reorganization of 2D fluorescence optical sections. By reconstructing the fluorescent signal in 3D and using the 3D surface to render the electrical tree channel (**Figure** **4**a–d), the development direction of the electrical tree is indicated by the arrow direction in Figure 4a. The wrinkles on the surface map illustrate the in situ morphological development of electrical trees inside the polymer. By adjusting the position of the section and setting it perpendicular to the development direction of the electrical tree (Figure 4b), the local area of interest and the local 3D topography of the electrical tree above the cross‐sectional plane at the third bifurcation point of the electrical tree can be observed and analyzed (Figure 4c). The occurrence of new electrical tree branches is also observed. Among them, one branch forms a complete discharge channel, while another branch forms discontinuous discharge channels (Figure 4d). Topography analysis determines that the discontinuous branches are thinner and have weaker fluorescence signals, and can therefore be regarded as a pre‐discharge channel. To verify the accuracy of the 3D fluorescence reconstruction results of electrical trees, we compared the results of different imaging modes, as shown in Figure S9, Supporting Information. The 3D fluorescent electrical tree morphology provides the possibility of understanding the overall structure and development direction of electrical trees, while providing a geometric support model for studying the development law and failure mechanism of electrical trees within polymers.

**Figure 5:**
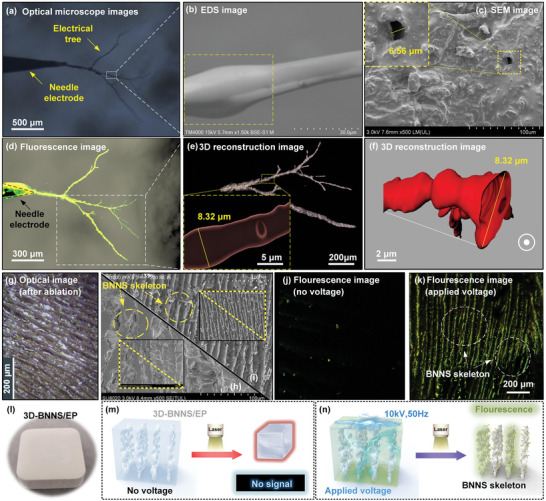
Comparison of results between traditional and fluorescence imaging methods. a) Optical microscope image of overall morphology of electrical tree. b) EDS image of the local electrical tree channel. c) SEM image of a cross‐section of the electrical tree channel. d) Fluorescence image of overall morphology of the electrical tree. e) 3D reconstruction image of local electrical tree and the 3D fluorescence morphology of electrical tree channel (yellow dotted box). f) 3D fluorescence imaging cross‐sectional view of electrical tree channel. g) Optical microscope image of skeleton morphology of BNNS after arc ablation. h) SEM image of cross‐section of the EP/BNNS skeleton. i) SEM image of cross‐section of the BNNS skeleton. j) Fluorescence image of BNNS skeleton (no voltage). k) Fluorescence image of BNNS skeleton (applied voltage). l) Physical map of 3D‐BNNS/EP. m,n) Schematic of comparison of the effect of voltage on fluorescence imaging results.

In traditional polymer internal damage detection or topography imaging, the polymer is required to be physically cut to obtain cross‐sectional information. To verify the accuracy of the damage inspection and 3D topography imaging methods proposed in this paper, the new method was compared with the traditional detection method for analyzing electrical damage channels inside the polymer (**Figure** **5**). First, we performed macroscopic, microscopic, and cross‐sectional imaging of the damaged area by optical microscopy (Figure 5a), energy dispersive spectroscopy (EDS) (Figure 5b), and scanning electron microscopy (SEM) (Figure 5c), respectively, to determine the morphology and size of the damaged area. Except for optical microscopy, all methods required destructive cutting of the detection samples. Judging by the overall macroscopic appearance of the damage, the fluorescence imaging method was superior in terms of clarity, recognition, and the display of detailed information (Figure 5d–f). Concurrently, the local channel of the electrical tree can be observed in the polymer body, through the section set in Figure 5e. The 3D fluorescence imaging image determined that the overall shape of the electrical tree channel was a 3D dendritic air gap connected by spherical fault points (Figure 5f), which is consistent with the EDS topography imaging results (Figure 5b). Finally, SEM (Figure 5c) and 3D fluorescence morphology measurements of single electrical tree channel pore size (Figure 5f) demonstrated that the diameters of the hole and electrical tree channel were 6.56 and 8.32 µm, respectively. The comparison results indicated that confocal fluorescence imaging technology could clearly image the morphology of the damaged area and accurately measure the diameter of the electrical tree channel. More importantly, this method is non‐destructive, and the internal morphology of the electrical tree channel could be observed without additional damage to the polymer body.

Further, to explore the wider application of this imaging method, we extended this method to cases where no damage occurred inside the polymer upon applying voltage. Hexagonal boron nitride nanosheets (h‐BNNS), which are thermally conductive skeleton‐like structures,^[^ ^35^, ^36^, ^37^ ^]^ are often present inside polymers and were tested using the three methods mentioned previously. In this study, when constructing the 3D boron nitride (BN) framework, the ice template method was used to induce the growth of ice crystals to form a 3D ordered framework (Figure 5k), and it was compounded with epoxy matrix by vacuum impregnation. Similarly, we compared the traditional imaging method with the new method. First, the BNNS skeleton was imaged by optical microscopy and SEM. The optical microscopy imaged the BN skeleton after arc ablation (Figure 5g), while SEM first required the composite material to be sliced. The two imaging methods demonstrate that the BNNS skeleton was successfully assembled into the EP polymer (Figure 5h,i). Confocal fluorescence imaging demonstrated that when no external electric field was applied to the polymer, no obvious fluorescence signal could be detected (Figure 5m,j), and when an external voltage of 50 Hz and 10 kV (electrode gap = 2 mm, *E* = 5 kV mm^−1^) was applied to the polymer, the voltage duration was 600 s (Figure 5n). During this time, we observed a very clear image of the BNNS skeleton under fluorescence imaging (Figure 5k and Movie S2, Supporting Information). This is consistent with the SEM detection results, which therefore proves the reliability of the new method.

### 2.3 Mechanism Study of Self‐Excited Fluorescence

We speculate that fluorescent substances are generated after an electrical tree occurs inside the dielectric polymer. However, this phenomenon has not been systematically studied and reported, and the mechanisms are still unclear. The newly formed substances cannot be determined owing to factors such as references and experimental equipment conditions. The fluorescence lifetime, which is the time it takes for a substance to enter the excitement state and reach the decay state, must also be considered each fluorescent substance has a specific fluorescence lifetime, and therefore, fluorescence lifetime analysis of the products in the channel was performed in this study (Figures S10 and S11, Supporting Information). This data can potentially be used to determine the quantity of the substances present and provide ideas for determining the substance composition.

**Figure 6:**
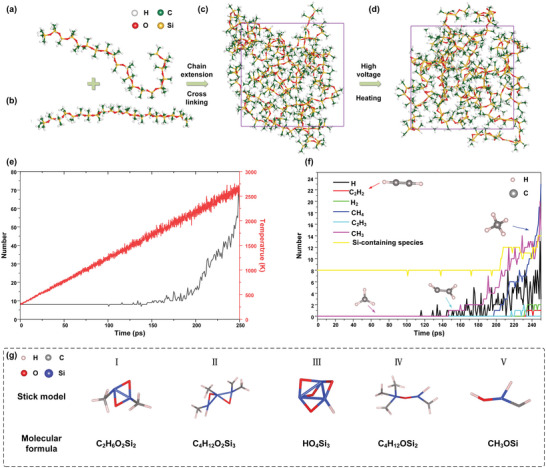
a) The molecular models of PMHS (C_38_H_118_O_19_Si_20_). C atoms shown in green, H in white, O in red, and Si in yellow. b) The molecular models of PMVS (C_46_H_126_O_19_Si_20_). c) The initial simulation cell of PMHS‐PMVS. d) The final simulation cell of PMHS‐PMVS after 250 ps molecular dynamics. e) Evolution of total number of molecules and temperature over time. f) Evolution of number of different species over 250 ps molecular dynamics simulation. g) Si‐containing species; I) C_2_H_6_O_2_Si_2_, II) C_4_H_12_O_2_Si_3_, III) HO_4_Si_3_, IV) C_4_H_12_OSi_2_, and V) CH_3_OSi.

The successful application of reactive force field (ReaxFF) in polymer pyrolysis shows that ReaxFF can reveal the reaction mechanism of polymer electrothermal process.^[^ ^38^, ^39^ ^]^ Therefore, we simulated the bond breaking and formation process of silicone gel under a strong electric field through ReaxFF to obtain the species that may cause the spontaneous fluorescence effect in the electrical tree channel. During calculation, four PMHS (**Figure** **6**a) and four PMVS molecule models (Figure 6b) were packed in a 30.5 × 30.5 × 30.5 Å^3^ periodic box at a density of 0.7 g cm^−3^ to generate a model system (Figure 6c). The model system was energy minimized and heated at a 9.4 K ps^−1^ heating speed from 300 to 2650 K for 250 ps. Incorporating electric field into the simulations was achieved by adding a force *F* = *qE* to each ion with an *E* = 5.0 × 10^−4^ V Å^−1^ *Z* direction electric field. It can be seen from Figure 6a–c that PMHS–PMVS is a linear polymer with Si—O bond as the main chain. Through calculation, we obtained the evolution curve of the total number of molecules and temperature with time in the cracking process (Figure 6e), and the evolution curve of the number of different substances in 250 ps (Figure 6f). It can be seen from the figure that high temperature accelerates the reaction speed of the polymer, and the presence of electric field promotes the cracking of the silicone gel (the species distribution table after the cracking of the silicone gel is given in the Supporting Information). The appearance of gas molecules such as CH_4_, H_2_, and free radicals such as H^+^ and CH_3_^+^ marks the beginning of pyrolysis. With the increase of energy, the appearance of C_2_H_2_ marks the occurrence of high‐energy discharge in the silicone gel. With the occurrence of electric cracking, the cracking further produced Si‐containing substances (Figure 6g). Through the analysis of the product results, it is found that there are cyclic siloxanes in the pyrolysis products (Figure 6g(I–III)). We believe that these products are the possible reasons for the spontaneous fluorescence of the tree channels. When Si—O is bonded into a ring,^[^ ^40^, ^41^ ^]^ it has a certain aggregation‐induced luminescence ability, and can effectively overcome the aggregation‐quenching effect of the luminescent group and improve the photostability of the material fluorescence. In addition, various functional groups in the aggregates form compact clusteroluminogens under the action of space electronic communication and double hydrogen bonds with Si—O as bridges, which facilitates in building larger electron delocalization system of the molecule as a whole and improves the luminescence performance.^[^ ^42^ ^]^ Therefore, we believe that cyclic siloxanes exhibit typical clusterluminescence, and the formation of clusters is the fundamental reason for self‐excited fluorescence. Their unique luminescence is essentially derived from cluster excitons.^[^ ^43^, ^44^ ^]^ In other words, it can be considered that cluster leads to the luminescence of unconventional systems. This result can potentially be used to determine the quantity of the substances present and provide ideas for determining the substance composition.

## 3 Conclusion

In conclusion, this study proposed a 3D imaging method based on material fluorescence self‐excitation with the aim to solve the issue of micro‐scale damage imaging in polymers. In this study, we report for the first time that the internal electrical tree channel of polymers subjected to an electrical field can generate self‐excited fluorescence. Furthermore, the self‐excited fluorescence was detected in the electrical tree region of the polymer by exposing a laser, and therefore the 3D fluorescence morphology of micro‐scale damage channels was obtained by external laser‐induced excitation of fluorescence in the channel area of the polymer. The fluorescence intensity accurately reflected the severity of internal damage, and the in situ fluorescence morphology characterized the 3D structure of the electrical tree. Finally, the cracking process of silicone gel under electric field was calculated by ReaxFF simulation, and it was found that the cyclic siloxanes compound generated after cracking was a possible reason for the spontaneous fluorescence of electric tree channel. Therefore, this method could solve the previously reported issues regarding high‐precision 3D imaging, by providing a method for non‐destructive testing, and in situ reconstruction of micro‐scale damage inside polymers. Furthermore, to the best of our knowledge, this method realizes the accurate imaging of micro‐scale electrical tree degradation in silicone gel for the first time, and thus this discovery provides a novel research method for exploring the accurate spatial geometric morphology of internal damage in dielectric polymers, and demonstrates the great potential regarding the application of in situ non‐destructive imaging in polymers.

## 4 Experimental Section

### Synthesis of Silicone Gel

The process of preparation of the sample silicone gel was as follows: First, components A (vinyl silicone oil) and B (hydrosilane) were fully and evenly mixed in a beaker, and the mixture was stirred for 15 min to ensure the full reaction of the two reagents. The mixture ratio had a ratio of components A to B of 1:1, and the temperature was maintained at 20 °C. The mixture was placed in a vacuum drying oven and degassed under −0.08 MPa for 5 min, during which the mold was heated to 140 °C and the release agent was sprayed to facilitate the subsequent removal of the sample from the mold. The reagent was poured into the mold after degassing and the sample was cured at 90 °C for 2 h and then at 20 °C for 8 h.

### Preparation of 3D‐BNNS/EP

First, a certain mass of exfoliated BNNS was added to the sodium carboxymethylcellulose (SCMC) binder solution, followed by ball milling at a rotation speed of 300 rpm for 2 h. Then, the homogeneous BNNS aqueous slurry was obtained by magnetically stirring the mixture of BNNS/SCMC suspension dispersion at 80 °C for 5 h. The slurry was then poured into a Teflon mold that adhered to a copper rod and was immersed in liquid nitrogen to perform directional freezing. During this process, the suspended BNNS would be expelled from the growing ice crystals, forming wall‐patterned hierarchical structures. After that, the frozen sample was sublimated by vacuum freeze‐drying at −80°C/0.5 Pa for over 48 h to get 3D BNNS skeleton. Subsequently, the dried 3D eGF and BNNS skeleton was immersed in the mixture of DGEBA, MTHPA, and DMP‐30 (100:80:2) at 60 °C for 2 h. Then, the sample was transferred into a vacuum oven and further impregnated under the assistance of vacuum at 60 °C for 4 h. The completely impregnated composite was then pre‐cured at 90 °C for 2 h and post‐cured at 110 °C for 2 h. Finally, the cured sample was stepwise cooled down to the room temperature to obtain 3D‐BNNS/EP composite.

### Simulation Method

Packmol was used to build the initial model system.^[^ ^45^ ^]^ LAMMPS was used to perform the molecular simulations.^[^ ^46^ ^]^ The ReaxFF simulations used the same parameters for C/H/O/Si interactions employed by Chenoweth et al.^[^ ^47^ ^]^ During the ReaxFF NVT molecular dynamics simulation, a time step of 0.1 fs was used to ensure the stability of the simulation. The temperature was controlled using the Nosé–Hoover thermostat with a relaxation time of 10 fs. Equations of motions were integrated using the velocity–verlet method. During the ReaxFF molecular dynamics simulation, the atomic coordinates were saved for post‐analysis.

## Conflict of Interest

The authors declare no conflict of interest.

## Supporting information

Supporting Information

Supplemental Movie 1

Supplemental Movie 2

## Data Availability

Research data are not shared.
