# Supplementary material for: Nondestructive 3D Imaging of Microscale Damage inside Polymers—Based on the Discovery of Self‐Excited Fluorescence Effect Induced by Electrical Field
Source: Adv Sci (Weinh). 2023 Jun 28;10(25):2302262. doi: 10.1002/advs.202302262 (PMC10477876; doi:10.1002/advs.202302262)
Supplement: Supplementary file 1 — Supporting Information [file ADVS-10-2302262-s003.pdf]

ADVANCED SCIENCE
Open Access

Supporting Information

for *Adv. Sci.*, DOI 10.1002/advs.202302262

Nondestructive 3D Imaging of Microscale Damage inside Polymers—Based on the Discovery of Self-Excited Fluorescence Effect Induced by Electrical Field

Wenxia Sima, Xinyu Tang, Potao Sun, Zhenkun Sun, Tao Yuan, Ming Yang, Chun Zhu, Zeyan Shi and Qin Deng*

Supporting Information

Nondestructive 3D Imaging of Microscale Damage Inside Polymers—Based on the Discovery of Self-Excited Fluorescence Effect Induced by Electric Field

Wenxia Sima[1]†, Xinyu Tang[1]†, Potao Sun[1]*, Zhenkun Sun[2], Tao Yuan[1], Ming Yang[1], Chun Zhu[2], Zeyan Shi[1], Qin Deng[3]

1. State Key Laboratory of Power Transmission Equipment and System Security and New Technology, Chongqing University, Chongqing 400044, China.
2. Key Laboratory of Energy Thermal Conversion and Control, Ministry of Education, School of Energy and Environment, Southeast University, Nanjing 210096, China.
3. Analytical and Testing Center, Chongqing University, Chongqing 400030, China.

†. These authors contributed equally: W. Sima, X. Tang.

*Corresponding author: sunpotao@cqu.edu.cn

Electrical trees in the polymers

Dielectric polymers age over time due to physical and electrical damage caused by the high mechanical and electrical stress to which the insulating material is subjected. Furthermore, the dielectric polymers become more sensitive to electrical breakdown due to material degradation and electronic avalanches, which makes unavoidable contamination or defects in dielectric materials. All of those age and defects can concentrate electrical stress and cause partial discharges in the microscopic regions, which leads to accelerating damage inside the material, disrupt the material structure and form a dendritic structure with a similar shape of lightning, called the electrical tree.

Generally, the development of electric trees can be divided into four stages as shown in Fig. S1. Electric trees are initiated at highly divergent electric field points such as metal particles and air gaps, and they grow slowly over a long period of time accompanied by tips dividing, and finally enter the runaway phase until breaking down.

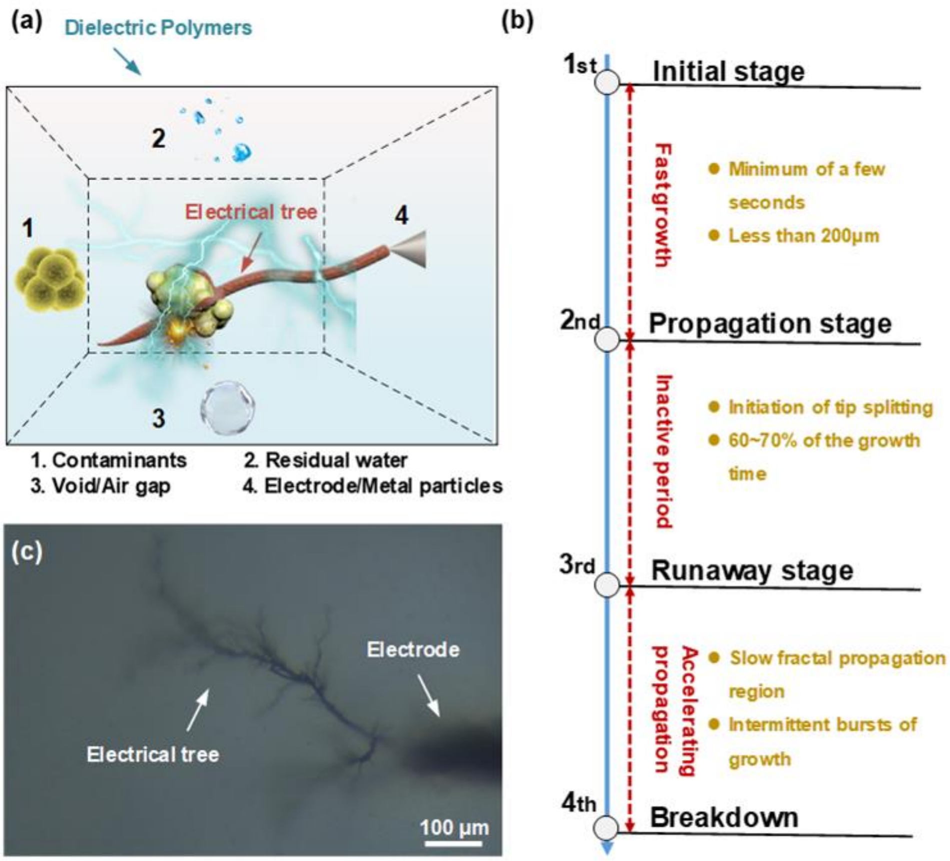

Figure. S1. Electrical tree in dielectric polymers. (a) The main factors that cause defects in the electrical tree of polymers. (b) The main developmental stages of electrical tree. 1st: initial stage - rapid development, usually less than 200 μm in length, 2nd: development stage - slow development, accounting for more than 60% of the entire electrical tree growth process, 3rd: accelerated development with tips dividing, 4th: polymer occurs breakdown, insulation failure. (c) Optical microscopy image of polymer electrical tree.

XCT Imaging Result

In the article, d2 omnipotent micro-nano focus CT system is utilized to detect the internal damage structure of the material. The sample is rotated 360° within the detection range and meanwhile irradiated. All two-dimensional projected images are collected angle by angle, which are rebuilt by computer to obtain three-dimensional data. All Test parameters and test results are shown in Table S1 and Figure. S2. Those results show that it is incompetent to detect obvious damage signals inside the polymer via XCT.

Table S1. Technical parameters and specifications

Technical Parameters	Specifications
Testing Equipment	diondo d_2 all-round micro-nano focus CT detection system
Voltage	90 kV
Current	90 μA
FDD	802 mm
FOD	12 mm
Integration time	2000 ms
Number of projection sheets	1860
Resolution	0.002 mm

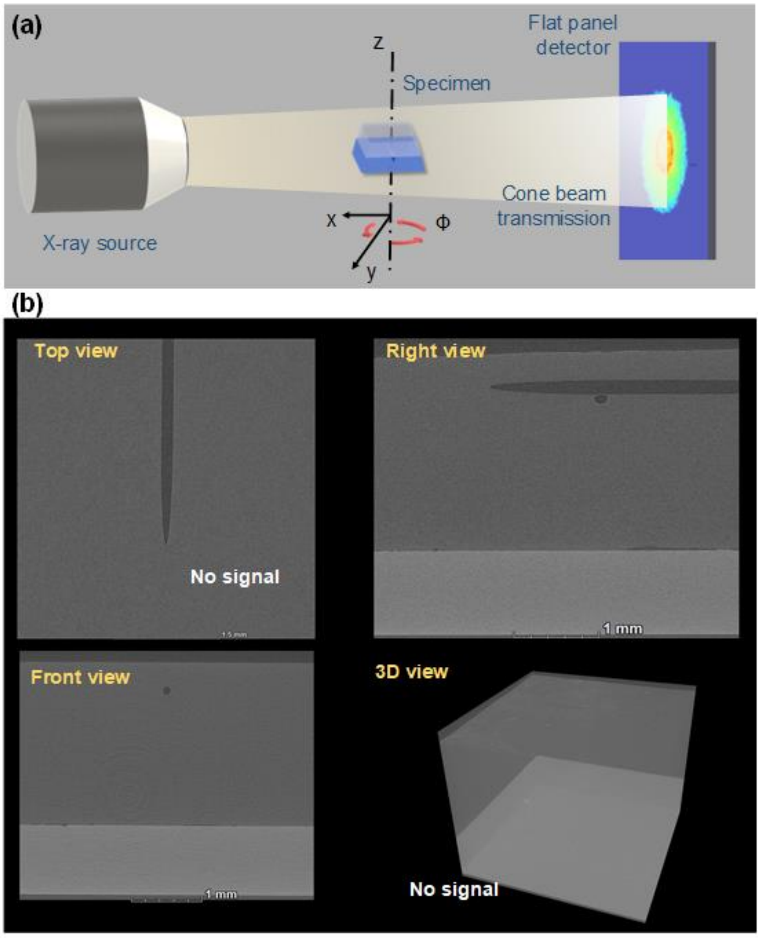

Figure. S2. XCT Imaging Results of Damage Inside the Polymer (a) Schematic of the Basic Principle of XCT Imaging (b) XCT Imaging Results and Tri-View.

Imaging principle of fluorescence microscopic technology

The Jablonski diagram shown in Fig. S3 describes this process. Generally, fluorescence luminescence conforms to Stokes shift, which means molecules absorb a short-wavelength photon and then emit a long-wavelength photon. So, the energy of fluorescence is less than that of absorbed radiation. As shown in Figure. S3, S_0 and S_1 respectively represent the ground state and the first electron excited singlet state of the molecule. T_1 represents the first electron excited triplet state of the molecule. The substance molecules transition from S_0 to S_1 after absorbing the incident light with a specific wavelength. During the molecular transition process, collision and friction induce energy loss, which leads to molecules returning to S_0 from S_1. Meanwhile, energy is released the form of luminescence which generates fluorescence.

The fluorescence signal was reflected back to the dichroic mirror through the original incident optical path. The returning signal was collected and amplified using the photomultiplier tube at the detection pinhole, and the optical signal was converted into an electrical signal. The fluorescence image of the tree channels was then obtained by inverse calculation of the electrical signal obtained in the test. By employing the coupled technique of pinhole and point light sources to filter out stray light, significant improvements were observed in the contrast and signal noise ratio of the image, as well as in the spatial resolution of the system. During the imaging process, slice imaging was realized by scanning the sample point by point, thereby achieving 3D high-resolution imaging of the polymer.

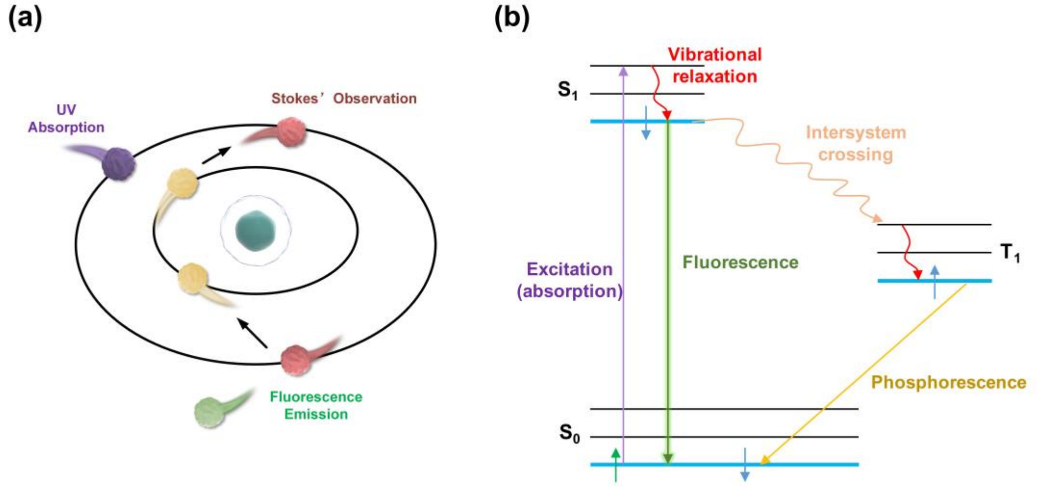

Figure. S3. Fluorescence excitation process. (a) Principle of fluorescence imaging of material damage (b) Jablonski energy level diagram. The electronic states are represented by horizontal lines, where the thick lines represent the corresponding vibrational ground states. Transitions within and between electronic states can be either nonradiative (indicated by red arrows) or radiative (indicated by green and yellow arrows) occurring at different time. Fluorescence occurs on the nanosecond scale. In most cases, the excitation and emission wavelengths produce Stokes shifts. S0: ground state; S1: the first electron excited singlet state of the molecule; T1: triplet energy state.

The effect of excitation wavelength on fluorescence imaging

The experiments show that the electrical damage channels inside the silicone gel can release fluorescent signals under the excitation of lasers with various wavelengths. In order to determine the optimal excitation wavelength, the local electrical trees in the silicone gel are excited by different wavelengths of laser. Figure. S4 shows the fluorescence imaging results of the different imaging modes. The scanning pixel in the Z-axis direction is improved to 0.1 μm when imaging the local area of electrical trees. The comparison of 2D optical sections in different imaging modes indicates that the fluorescent image signal cannot be detected in the bright field, as shown in Figure. S4(a). Yet the fluorescence signals of the tree channel are detected under the excitation wavelengths of 488 nm and 596 nm, as shown in Figure. S4(d) and Figure. S4(e), respectively. To obtain the best imaging quality, the laser of 488 nm wavelength is chosen as excitation source to perform fluorescence imaging on the tree channel.

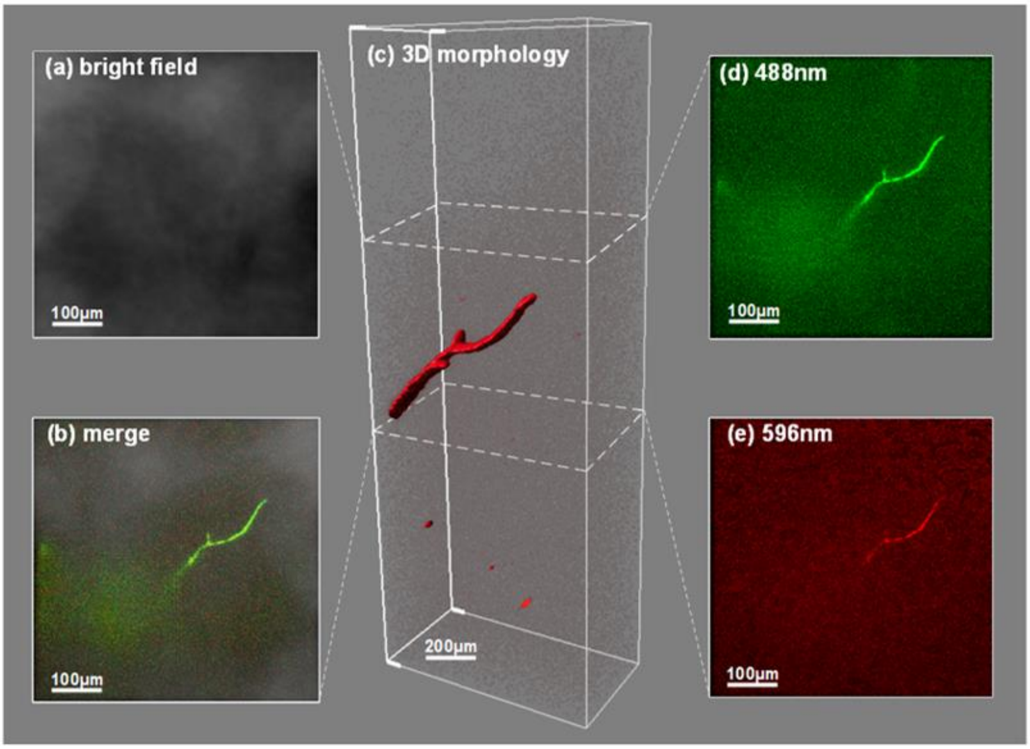

Figure. S4. Comparison of different imaging modes (a) Brightfield imaging mode (b) Merged imaging mode (c) 3D surface image of local electrical tree (d) 488nm excitation fluorescence imaging mode (e) 596nm excitation fluorescence imaging mode.

Table S2 Scanning parameters in the XYZ direction

**Dimension**	**Logical Size**	**Physical Length**	**Start Position**	**End Position**	**Pixel Size**
X	1024	1550μm	0μm	1550μm	1.515μm
Y	1024	1550μm	0μm	1550μm	1.515μm
Z	69	203.89μm	140.8μm	344.69μm	1.998μm

Experimental platform and samples

In this experiment, a typical needle-plate electrode configuration was used to accelerate the electrical degradation of the polymer. Copper needle electrodes were embedded in the samples, and a high-frequency pulsed electric field was applied across the electrodes to simulate electrical tree micro-defects (Fig. S5.).

The copper needle is 40 mm long, 1.2 mm in diameter, and has a tip radius of 0.12mm. The gap distance between the needle electrode and the ground electrode (plate electrode) is 2mm, as shown in Figure. S5. (b). The electric field across the needle-plate electrodes is applied by an all-solid-state nanosecond pulse generator, as shown in Figure. S5. (a). Specifically, the pulse amplitude is 0~15kV (continuously adjustable), pulse width is 100~1000ns, repeatable frequency is 1Hz~10kHz, rising edge 30ns and falling edge less than 50 ns. The initiation and development of electric trees are observed via HD 4K industrial camera (150 times magnification, real-time frame rate 60FPS) during experiment.

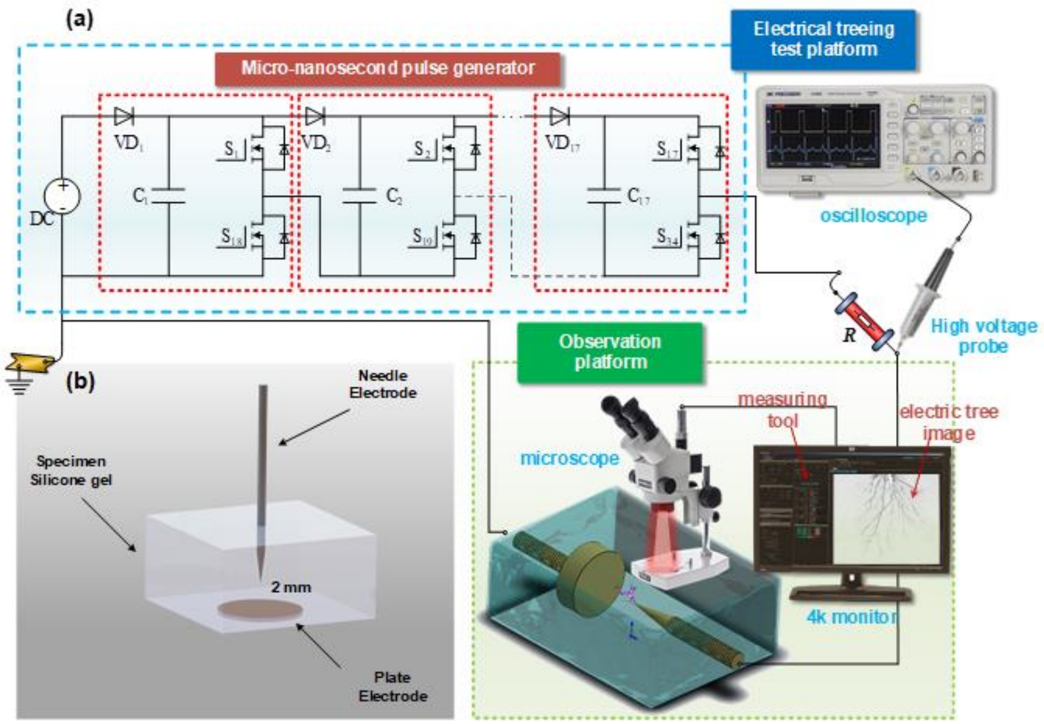

Figure. S5. Electrical tree experimental platform via high-frequency pulse (a) The circuit of the all-solid-state nanosecond pulse generator and the schematic diagram of the experimental circuit (b) The schematic of the needle-plate electrode parameters.

Fluorescence signal localization map of the electrical trees

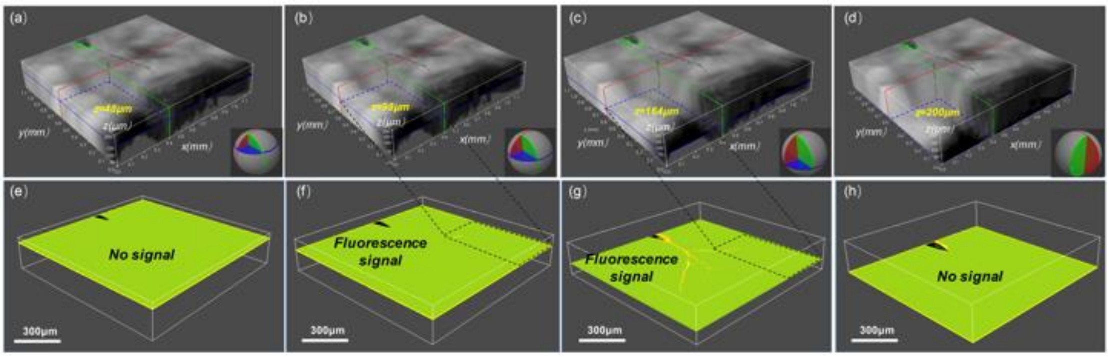

Figure S6. Fluorescence signal localization map of the electrical tree (a) z=48 μm, no fluorescence signal is observed. (b) z=98 μm, fluorescence signal is observed. (c) z=164 μm, fluorescence signal can be clearly observed (d) z=200 μm No fluorescent signal is observed. (e)-(h) correspond to (a)-(d) 2D in vivo fluorescence optical sections at Z-axis depths.

Fluorescence intensity of 2D slices in the electrical tree area

Figure. S7 is an easy 3D electrical tree image formed by the projection of all fluorescent signals on the plane. It is composed of optical sections of different focal planes and contains considerable spatial information. The fluorescence topography and intensity information in different focal planes can be obtained by decomposing this information (Figure. S8). Fig. S6b illustrates partial 2D fluorescence slice imaging in the framed area, which is a major advantage of confocal fluorescence microscopic technology as it enables in vivo scanning of the entire sample and any area. A total of 24 fluorescence slices were achieved and the number of light slice decomposition is related to the Z-axis resolution.

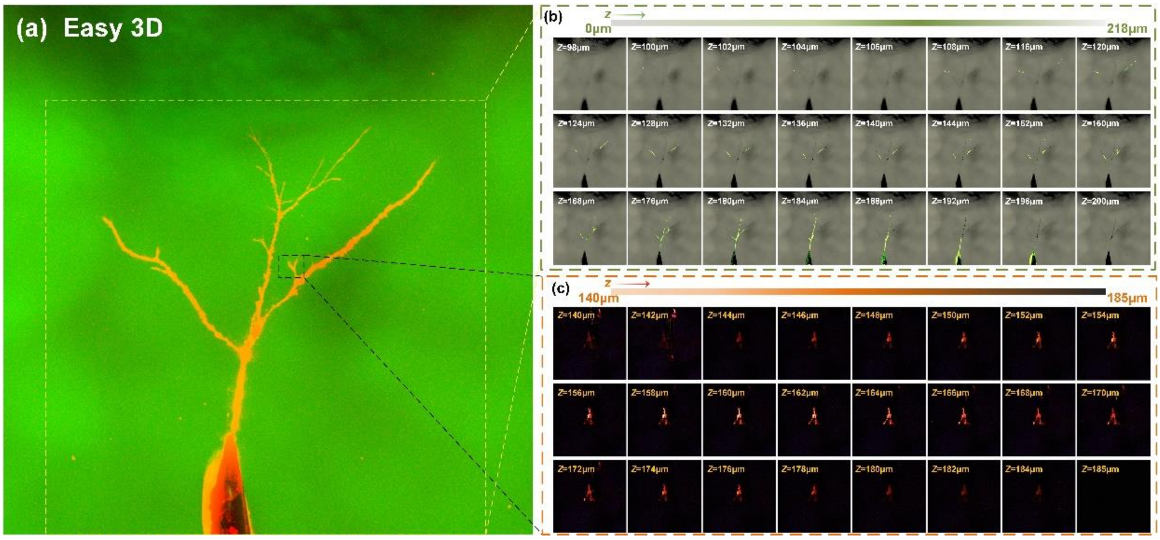

Figure. S7. Electrical tree self-excitation fluorescence images in different scanning planes and schematic illustration of local channel reconstruction. (a) Superimposed projection image of the electrical tree fluorescence signal plane (Easy 3D image). (b) 2D fluorescence imaging light images at different Z-axis depths ranging from 98–200 μm. (c) Local 2D fluorescence imaging light images at different Z-axis depths. The signal range is 140–185 μm and the fluorescence signal appears at a depth of 144 μm, is strongest at 160 μm, and disappears at 185 μm.

Fluorescence intensity is a key advantage to reflect fluorescence imaging variability. During the imaging process, the severity of damage to the interior of the polymer by the electrical tree channel can be determined by fluorescence intensity. Fluorescence

intensity distribution of 2D slices in the tree area with different Z-axis depths is quantified and compared to the region in the dotted box, as shown in Figure. S8. (a) ~ (c), which shows fluorescence intensity is positively correlated with the area of the tree region. The fluorescence intensity distribution of different Z-axis depths is different. With the increase of Z-axis depth, the tree area becomes more concentrated, and the fluorescence signal becomes stronger. This can determine the location of the electrical tree in the polymer, as well as the development direction and branching range of the tree inside the polymers, which can provide a geometric quantitative numerical basis for the analysis of the electrical tree development mechanism.

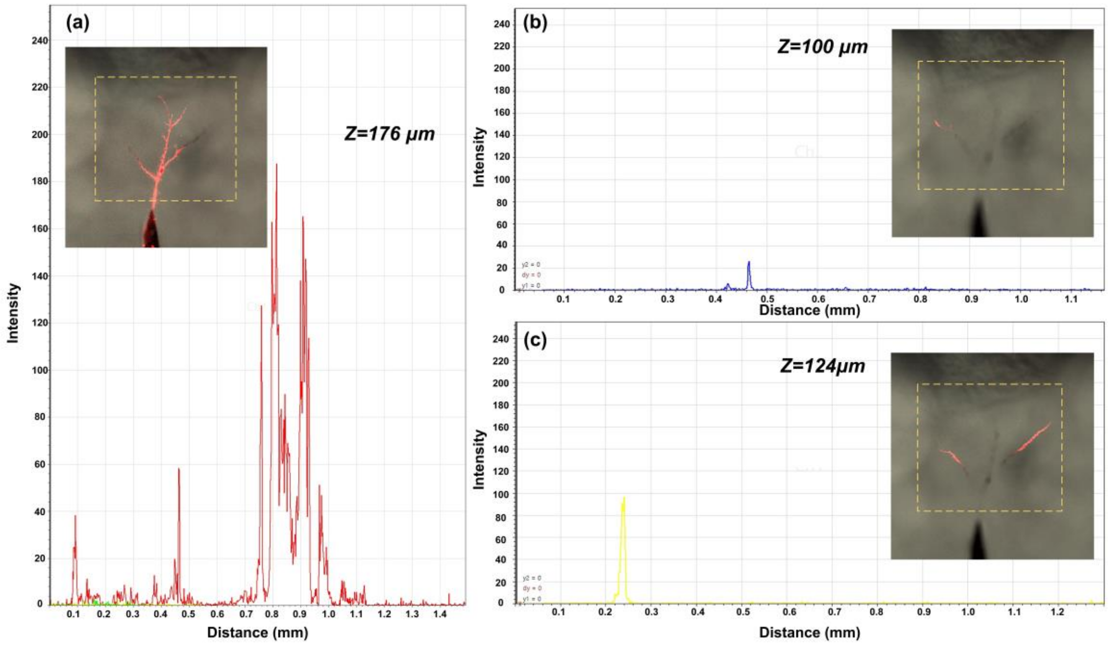

Figure. S8. Fluorescence intensity distribution of 2D slices electric tree in the yellow box region along the vertical upward direction at the needle electrode (a) Fluorescence signal intensity distribution at Z=100μm (b) Fluorescence signal intensity at Z=124μm (c) Fluorescence signal intensity at Z=176 μm.

Electrical tree images under different imaging modes

Figures S9(a) - (c) show the Easy 3D, 2D fluorescence, and 2D optical slices of the electrical trees, respectively. We located the local area of the electrical tree in Figure 4 (d) and found that no signal in this area under the three imaging modes, and it exhibited discontinuous electrical tree morphology. This may be caused by partial discharge in the silicone gel under the strong electric field, and the electric branch channel shrinks when the external electric field is removed. This is because the electrical tree inside the silicone gel is mainly composed of air passages and bubbles, which promotes the generation of hollow crack channels under the dual action of air pressure and electrical stress. When the external electric field disappears, the gas pressure in the channels rapidly decreases [1] - [2].

Furthermore, we validated the accuracy of the electrical tree region in Figure 4 (d) through 3D imaging. Figure S9 (d) shows the 3D in situ fluorescence imaging of electrical trees. The areas with electrical damage have fluorescence signals, while the areas without damage have no fluorescence signals. Figure S9 (f) shows the 3D reconstructed morphology of fluorescent electrical trees, in order to verify the accuracy of the reconstructed morphology. We superimposed the fluorescence signal with the reconstructed morphology, as shown in Figure S9 (e). The superposition results indicate that the in-situ fluorescence signal effectively fills the reconstructed morphology of the electrical tree channels, which proves the accuracy of the reconstruction. And from Figures S9 (d) - (f), it is shown that the electrical tree region in Figure 4 (d) is discontinuous, not due to the discontinuity caused by the reconstruction process, but

because there is no electrical tree damage in this region. Since this discontinuous phenomenon occurs at the end of the electrical tree channel, it may also be the pre-discharge channel inside the silicone gel. This pre-discharge phenomenon was also mentioned in the literature [3].

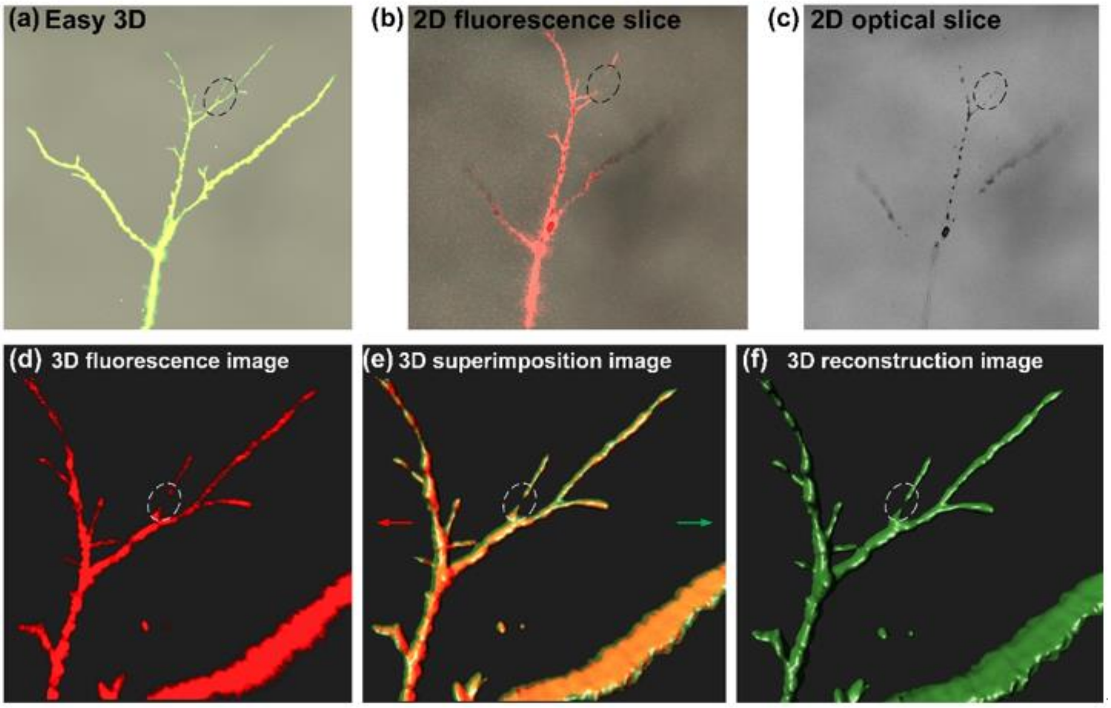

Figure. S9. Electrical tree images under different imaging modes (a) Easy 3D (b) 2D fluorescence slice (c) 2D optical slice (d) 3D fluorescence image (e) 3D superimposition image (f) 3D reconstruction image

Fluorescence lifetime imaging of electrical tree areas

The fluorescence lifetime defines as the time that takes for the fluorescence intensity to decay to 1/e of its maximum value. The fluorescence lifetime reflects the average lifetime of the S_1 excited state of the fluorescent molecule. Different substances have different fluorescence lifetimes. The decay curve has non-single exponential characteristics, and the effective fluorescence lifetime can be calculated according to formula (1).

$$\tau_{\mathrm{exp}} = \int I(t)dt / I_P \quad (1)$$

where I_P is the peak intensity, $I(t)$ is a function of fluorescence intensity and time, and t is time. By testing and calculating the fluorescence signal in Figure. S10, it is found that the fluorescence lifetime of the fluorescence signal in the figure is between 0 and 4.02ns. It is found that three substances with self-excited fluorescence effect appeared in the electrical tree channel, and the fluorescence lifetime of each substance is calculated, as shown in Figure. S11. It is found that the strongest imaging signal with the fluorescence lifetime peak is at 3.4ns, which can be considered that the most fluorescent substances are at this lifetime.

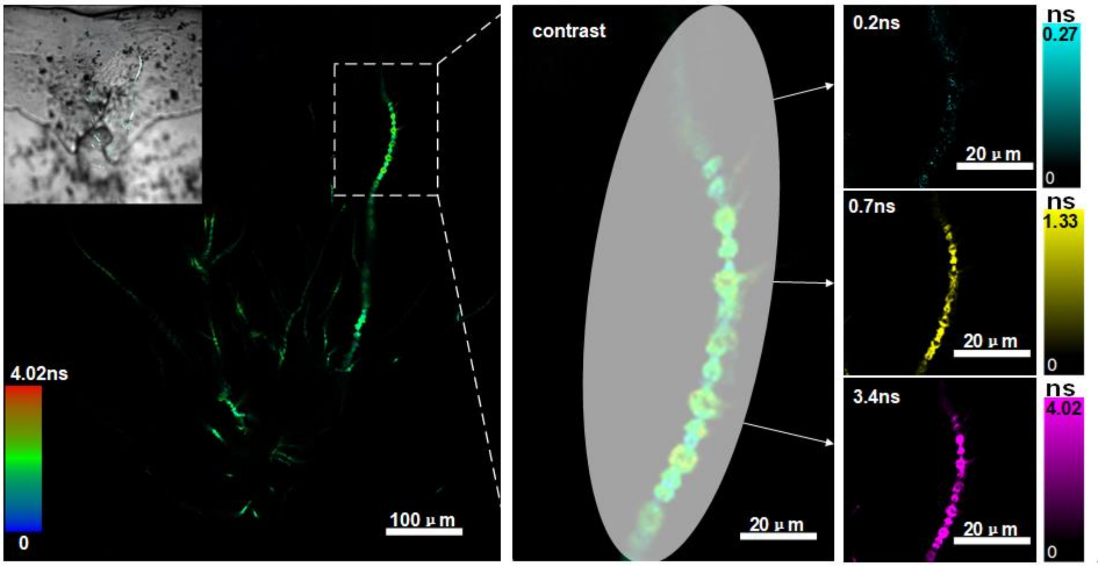

Figure. S10. Fluorescence lifetime diagram of electrical tree Left: Composite graph of fluorescence lifetime of electrical tree, middle: Enlarged image in dotted box right: Decomposed graph of fluorescence lifetime in electrical tree channel, the color bar corresponds to the life cycle of the fluorescent substance, expressed in ns.

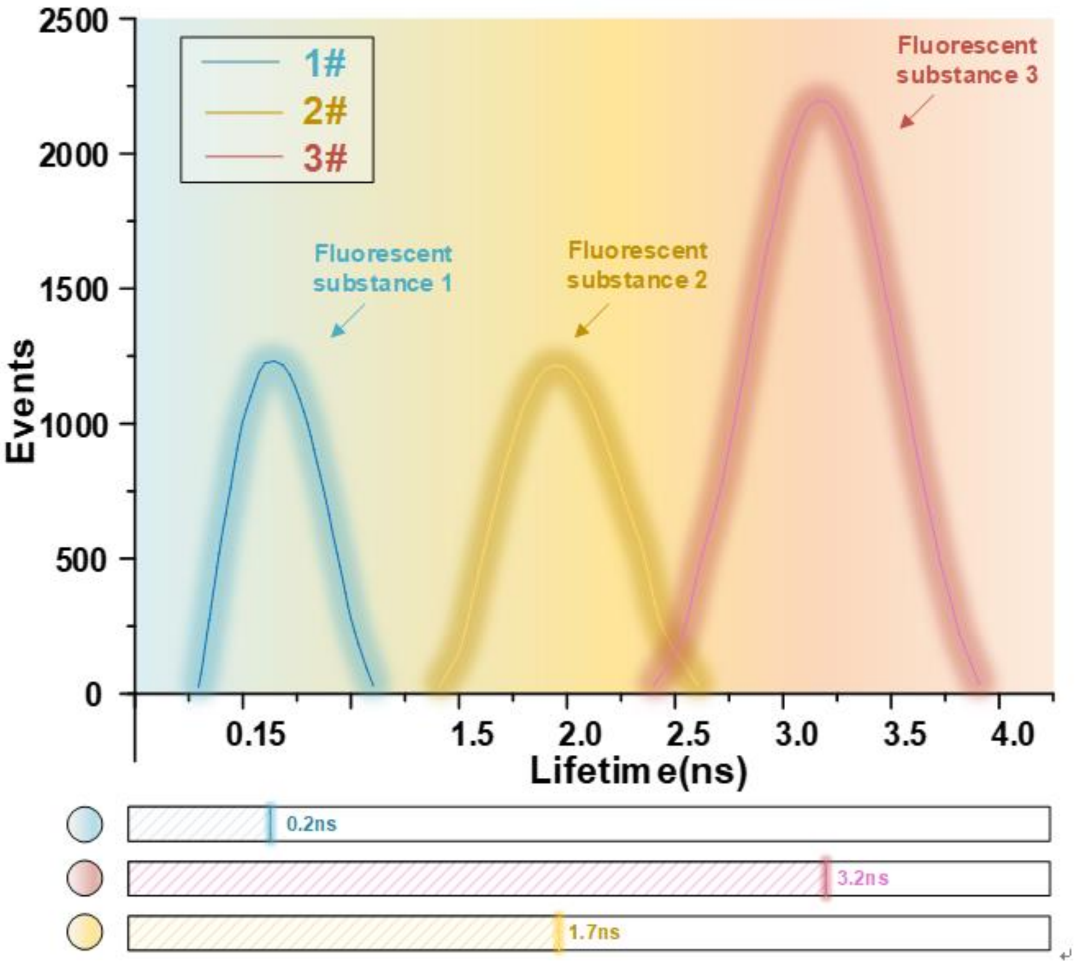

Figure. S11. Fluorescence lifetime graph of Gaussian distribution of fluorescent substance in electrical tree channel.

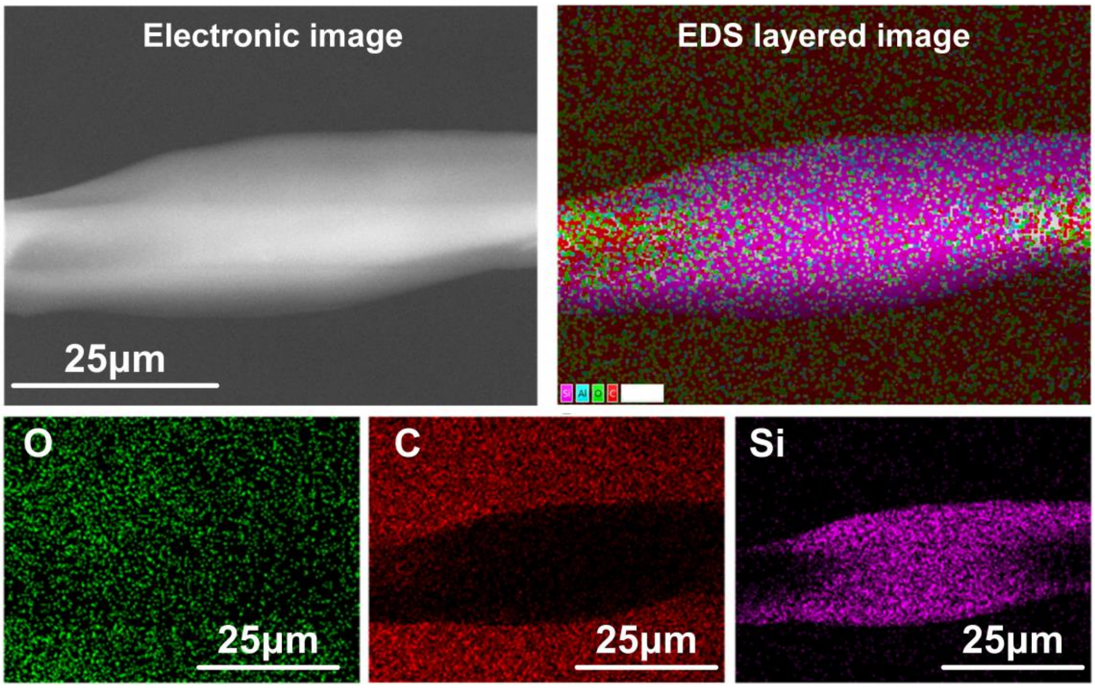

Figure. S12. Distribution of main elements in the electrical tree channel.

Table S3. The resulted species after 250 ps molecular dynamics simulation.

Species	Number
CH_3	15
CH_4	23
C_2H_2	1
C_2H_6	2
CHO	1
CH_3OSi	1
$C_2H_6O_2Si_2$	2
$C_{24}H_{70}O_{15}Si_{15}$	1
$C_{24}H_{72}O_{15}Si_{16}$	1
$C_{27}H_{80}O_{16}Si_{17}$	1
$C_{30}H_{91}O_{17}Si_{18}$	1
$C_{37}H_{98}O_{18}Si_{19}$	1
$C_4H_{12}OSi_2$	1
$C_4H_{12}O_2Si_3$	1
$C_{44}H_{119}O_{20}Si_{22}$	1
$C_{46}H_{122}O_{19}Si_{20}$	1
$C_{46}H_{125}O_{19}Si_{20}$	1
H	3
H_2	2
HO_4Si_3	1

References

[1] M. Sato, A et al, "Surface discharges in silicone gel on AlN substrate," IEEE Transactions on Dielectrics and Electrical Insulation, vol. 23, no. 1, pp. 494-500, February 2016, doi: 10.1109/TDEI.2015.005412.

[2] L. M. Salvatierra et al., "Self-healing during electrical treeing: A feature of the two-phase liquid-solid nature of silicone gels," in IEEE Transactions on Dielectrics and Electrical Insulation, vol. 23, no. 2, pp. 757-767, April 2016, doi: 10.1109/TDEI.2015.004813.

[3] Pallon LK et al, "Three-Dimensional Nanometer Features of Direct Current Electrical Trees in Low-Density Polyethylene," in Nano Lett, vol. 8, no. 17, pp. 1402-1408, Mar 2017, doi: 10.1021/acs.nanolett.6b04303.
